# Oxidative stress and ROS metabolism via down-regulation of sirtuin 3 expression in *Cmah*-null mice affect hearing loss

**DOI:** 10.18632/aging.100800

**Published:** 2015-08-29

**Authors:** Deug-Nam Kwon, Woo-Jin Park, Yun-Jung Choi, Sangiliyandi Gurunathan, Jin-Hoi Kim

**Affiliations:** Department of Animal Biotechnology, Konkuk University, Seoul 143-701, Republic of Korea

**Keywords:** hearing loss, CMP-N-acetylneuraminic acid hydroxylase, ROS metabolism, mitochondria dysfunction, oxidative stress

## Abstract

CMP-Neu5Ac hydroxylase (*Cmah*) disruption caused several abnormalities and diseases including hearing loss in old age. However, underling molecular mechanisms that give rise to age-related hearing loss (AHL) in *Cmah*-null mouse are still obscure. In this study, *Cmah*-null mice showed age-related decline of hearing associated with loss of sensory hair cells, spiral ganglion neurons, and/or stria vascularis degeneration in the cochlea. To identify differential gene expression profiles and pathway associated with AHL, we performed microarray analysis using Illumina MouseRef-8 v2 Expression BeadChip and pathway-focused PCR array in the cochlear tissues of *Cmah*-null mouse. Pathway and molecular mechanism analysis using differentially expressed genes provided evidences that altered biological pathway due to oxidative damage by low expressed antioxidants and dysregulated reactive oxygen species (ROS) metabolism. Especially, low sirtuin 3 (*Sirt3*) gene expressions in *Cmah*-null mice decreased both of downstream regulator (*Foxo1* and *MnSod*) and regulatory transcription factor (*Hif1α* and *Foxo3a*) gene expression. Taken together, we suggest that down-regulation of *Sirt3* expression leads to oxidative stress and mitochondrial dysfunction by regulation of ROS and that it could alter various signaling pathways in *Cmah*-null mice with AHL.

## INTRODUCTION

N-glycolylneuraminic acid (Neu5Gc) is generated by hydroxylation of CMP-Neu5Ac to CMP-Neu5Gc, catalyzed by CMP-Neu5Ac hydroxylase (Cmah) [1, 2]. However, humans lack this common mammalian cell surface molecule, Neu5Gc, due to inactivation of the *Cmah* gene during evolution [3, 4]. Previous studies showed that disruption of *Cmah* gene affect various biological processes of animals [5, 6]. Our recent study has suggested that *Cmah*-null mice could be a useful model for studying diabetes with dysregulation of insulin/PI3K-AKT signaling [7]. In another study, *Cmah*-null mouse showed histological abnormalities of the inner ear occurred in older mice, which had impaired hearing [5]. On the other hand, it was reported in a study that, in a senescence-accelerated mouse, prone 8 (SAMP8) strain mouse shows premature hearing loss and cochlear degeneration repeating the processes noticed in human presbycusis (*i.e.,* strial, sensory, and neural degeneration) [8]. The molecular mechanisms correlated with premature age-related hearing loss (AHL) in SAMP8 mice comprises oxidative stress, distorted levels of antioxidant enzymes, and decreased activity of some complexes which as a result leads to chronic inflammation and prompting of apoptotic cell death pathways [8]. In another studies, rat F344/NHsd strain showed an increased mitochondrial DNA deletions in the cochlea at 6- and 9-months of age [9, 10]. As a result, mitochondrial DNA deletions or mutations causes impairment in mitochondrial function and eventually leads to buildup of cochlear oxidative stress and consequent cochlear degeneration and AHL [11]. Even though there is growing recognition that oxidative stress and reactive oxygen species (ROS) may be an important factor for underlying mechanism of hearing loss, the studies of molecular mechanisms of AHL is still infancy in *Cmah*-null mouse.

Recent study provided a possible mechanism of sirtuin 3 (Sirt3) in ROS and oxidative stress induced metabolism in mammals [12, 13]. Sirtuins are NAD^+^-dependent protein deacetylases [14]. They possess the ability to regulate life span in lower organisms and have turned up as immense cellular fate regulators and mammalian biology [15]. There are seven sirtuins in mammals; they demonstrate diverse cellular localization [16, 17]. Among them, Sirt3 localized in mitochondria stimulation results in activation of oxidative metabolism through deacetylation of numerous mitochondrial enzymes [18, 19]. Sirt3 has been found to predominantly regulate the production of ROS at the electron transport chain (ETC), further the detoxification of ROS via activation of antioxidant enzymes [20-25]. A current study implied that mitochondrial Sirt3 arbitrates the anti-aging effects of calorie restriction (CR) on AHL by promoting the glutathione-mediated mitochondrial antioxidant defense system in mice [23]. As CR is recognized to provoke Sirt3 protein levels in wild-type (WT) mice, there is a possibility that Sirt3 expression levels can mediate ROS management, mitochondrial integrity, and sensory function, at least in those neurons that constrain hearing [26-28]. CR-mediated aging retardation regulated by Sirt3 in mammal. However, CR failed and impaired to reduce oxidative damage in multiple tissues of *Sirt3* null mouse compare to control and it leads to the progression of AHL [29].

Despite growing interest in the relationship between loss of Cmah activity and age-related hearing loss, how genome-wide altered gene expression in the cochlear tissues of *Cmah*-null mouse affects hearing loss with aging remains unclear. In this study, we first explored differential gene expression profile in the cochlear tissues of *Cmah*-null mouse. We performed the functional enrichment analyses using annotations from both Gene Ontology (GO) and pathways. More importantly, we identified the pathway that related to the oxidative damage and ROS metabolism, supporting the pathogenesis hypothesis of age-related dysfunction. Finally, we analyzed the expression of their genes that involved Sirtuins, oxidative stress regulation, Wnt signaling, mitochondrial dysfunction identified by KEGG (Kyoto Encyclopedia of Genes and Genome) pathway analysis on microarray and a pathway-focused PCR array to address the mechanisms underlying AHL in *Cmah*-null mouse. In conclusion, we suggest a hypothetical model that down-regulated Sirt3 lead to oxidative damage and mitochondrial dysfunction by ROS and could alter various signaling pathways in *Cmah*-null mice with AHL. In this study, we propose a possible hypothetical model that down-regulated *Sirt3* lead to oxidative damage and mitochondrial dysfunction by ROS and it could alter various signaling pathways involved in *Cmah*-null mice with AHL.

## RESULTS

### Cmah disruption result in histological abnormalities in vestibular otoconial epithelia and the cochlear sensory epithelium, and neuronal cell loss of inner ears

**Figure 1:**
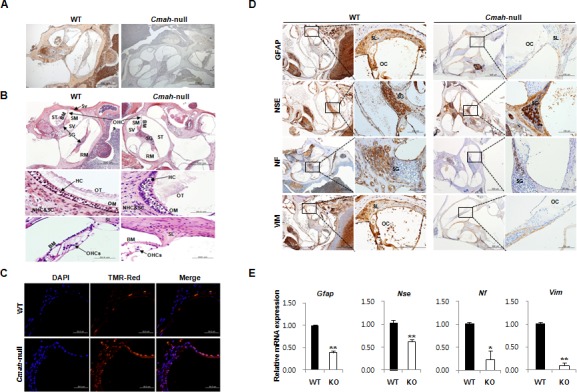
Age-related inner ear abnormality and neuron cell loss in *Cmah*-null mice (**A**) Analysis of Neu5Gc expression in cochlear tissues of WT- and *Cmah*-null mice by IHC using a chicken anti-Neu5Gc antibody. (**B**) Histological abnormality in inner ears of *Cmah*-null mice. Deposition of unusual and apparently a cellular material in the vestibular otoconial epithelia (upper). The area of the outer hair cells showed degeneration of the sensory cells throughout the cochlea in the cochlear sensory epithelium, (bottom). SV: scala vestibule, SM: scala media, ST: scala tympani, SG: spiral ganglion, BM: sasilar membrane, RM: reissner's membrane, Sv: stria vascularis, OT: otoconia, OM: otolithic membrane, NHC & SC: neuroepithelial hair cells and supporting cells, HC: hair cells, OHCs: outer hair cells, SL: Spiral lamina. (**C**) Apoptotic cell death in the cochlea of *Cmah*-null mice. TUNEL labeling performed on paraffin sections the cochlea from WT- and *Cmah*-null mice. (**D**) The expression of ganglion cells in cochlear tissues from WT- and *Cmah*-null mice by IHC. Rectangular box indicate a higher magnification images (5x) in left panel. GFAP: Glial fibrillary acidic protein, NSE: Neuron specific enolase, NF: Neurofilament, VIM: Vimentin. SL: spiral lamina, OC: organ of corti, SG: spiral ganglion. (**E**) mRNA expression pattern of neuronal cell markers in cochlear tissues from WT- and *Cmah*-null mice. RT-qPCR was used to measure the expression of *Gfap, Nse, Nf*, and *Vim* in the cochlea from WT- and *Cmah*-null mice.

*Cmah*-null mouse, like the previous observations, showed age-related hearing loss [5, 30]. To determine the lack of CMAH protein expression, we performed immunohistochemistry (IHC) in inner ear from WT- and *Cmah*-null mice. As shown in Fig. 1A, the Neu5Gc, which is a final product by hydroxylation of CMP-Neu5Ac to CMP-Neu5Gc by CMP-Neu5Ac hydro-xylase, was highly expressed in the WT, whereas the *Cmah*-null mice were completely deficient in inner ears. In histological analysis, 12 month- but not 5 week-old (Supplementary Fig. 1). *Cmah*-null mice had histological abnormalities in the vestibular otoconial epithelia and the cochlear sensory epithelium of inner ears (Fig. 1B). The apical surface of the vestibular otoconial epithelia contains unusual deposits of apparently a cellular material among the stereociliary bundles (Fig. 1B upper). Further, we observed degeneration of outer hair cell throughout the cochlea, with collapse of the outer organ of corti in the basal (high-frequency) turn of 12 month-old mice (Fig. 1B; bottom), whereas both of the cochlear and vestibular sensory epithelia of WT-mice appeared normal, even in older mice. Next, we performed the TUNEL assay to investigate the apoptotic cell death. The result shows significantly increased numbers of apoptotic cells in supporting cells of cochlear tissues from *Cmah*-null mice, compared to control (Fig. 1C).

In order to examine neuronal cell loss in *Cmah*-null mouse, we investigate the several neuronal cell-specific gene expressions in cochlear tissues. In this study, we examined glial fibrillary acidic protein (GFAP) expression for a neural injury marker or astrocyte marker, neurofilament (NF) expression for an axonal injury marker, vimentin (VIM) expression for a neural precursor cells marker, and enolase (NSE) expression for neuronal specific marker. In IHC, cochlear tissues of *Cmah*-null mice showed significant reductions in expressions of several neuronal cell markers such as GFAP, NSE, NF, and VIM in with respect to control mice (Fig. 1D). Reverse transcriptase quantitative real-time polymerase chain reaction (RT-qPCR) analysis also showed significantly down-regulated gene expression for same neuronal cell markers (*Gfap, Nse, Nf*, and *Vim*) compared to control (Fig. 1E), indicate that RT-qPCR results mimic IHC results.

### Differential gene expression profile in cochlea of *Cmah*-null mice

**Figure 2:**
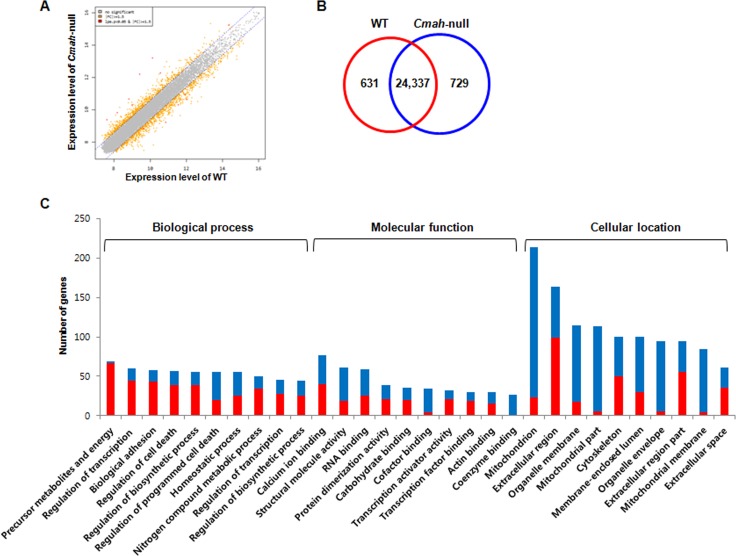
Gene expression profile in cochlea of *Cmah*-null mice (A) Scatterplot of normalized mRNA expression by microarray. Data are log2 normalized. Grey- and orange-spots indicate no significant genes and up- or down-regulated genes, with more than 1.5-fold change. (B) Venn diagram showing differential expression of genes in the cochlear tissues of *Cmah*-null mice. Numbers in red and blue Venn diagram present up- and down-regulated genes, respectively. (C) The differentially up- or down-regulated genes were clarified according to biological process, molecular function, and cellular component. GO classification significantly enriched with a Benjamini-Hochberg corrected P value < 0.05 among all differentially expressed genes with ≥ 1.5-fold expression between WT- and *Cmah*-null mice.

Expression profiles in cochlear tissues of WT- and *Cmah*-null mice (n=3, respectively) were compared using an Illumina MouseRef-8 v2 Expression BeadChip, which is composed of 25,697 probe sets for mouse mRNA. To analyze differential gene expression in WT- and *Cmah*-null mice, we focused on the gene expressions, which showed ≥1.5 folds changes with a significant p-value (p < 0.05). In this study, we performed scatter plot analysis in order to estimate global gene expression change. As shown in Fig. 2A, mRNA expression was normalized by the scatter plots: of 25,697 genes, 631 genes were up-regulated, whereas 729 genes were down-regulated in *Cmah*-null mice-derived cochlear tissues compared to control mice-derived cochlear tissues (Fig. 2B). Additional File 1 and 2 showed complete lists of these genes.

### Functional analysis of differentially expressed genes in cochlea of *Cmah*-null mice

To explore their biological functions, GO classification by functional annotation of the DAVID (Database for Annotation, Visualization and Integrated Discovery) tools categorize each genes with significant differential expression in cochlea of *Cmah*-null mice. Fig. 2C showed the top 10 enriched functional annotations in three categories according to GO classification. These functional annotations consisted of generation of precursor metabolites and energy (69 genes), regulation of transcription from RNA polymerase II promoter (60 genes), biological adhesion (58 genes), regulation of cell death (57 genes), regulation of biosynthetic process (55 genes), regulation of programmed cell death (55 genes), homeostatic process (55 genes), regulation of nitrogen compound metabolic process (50 genes), regulation of transcription (46 genes), regulation of biosynthetic process (44 genes) for biological process; calcium ion binding (77 genes), structural molecule activity (61 genes), RNA binding (59 genes), protein dimerization activity (39 genes), carbohydrate binding (35 genes), cofactor binding (34 genes), transcription activator activity (32 genes), transcription factor binding (30 genes), actin binding (30 genes), coenzyme binding (27 genes) for molecular function; mitochondrion (213 genes), extracellular region (163 genes), organelle membrane (115 genes), mitochondrial part (113 genes), cytoskeleton (100 genes), membrane-enclosed lumen (100 genes), organelle envelope (95 genes), extracellular region part (94 genes), mitochondrial membrane (84 genes), and extracellular space (61 genes) for cellular component.

**Figure 3:**
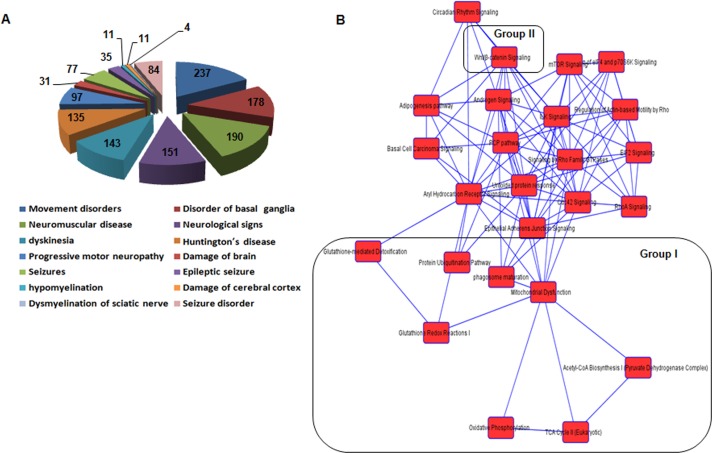
Functional categorization of neurological disease and network of canonical pathways objected by IPA using genes differentially expressed in the cochlea of *Cmah*-null mice (**A**) Pie chart analysis of the disease or functions annotation for neurological disease. (**B**) The network of canonical pathways highlights the relationships between pathways and classifies the functional groups by IPA analysis. Group I and II represent oxidative stress and mitochondrial dysfunction-related signaling (Group I), and Wnt/β-catenin signaling (Group II), respectively.

Next, we examined differentially expressed genes that are enriched in pathways by KEGG pathway analysis. Table 1 show the 11 most significantly enriched pathways. Of note, most of them are closely associated with oxidative damage and metabolism, supporting the pathogenesis hypothesis of age-related dysfunction. These results are consistent with prior knowledge of AHL [5, 11, 29]. In addition, ingenuity analysis identified more putative diseases and disorders in cochlear tissues of *Cmah*-null mice. These are as follows: neurological disease, psychological disorders, skeletal and muscular disorders, infectious disease, and hereditary disorder (Table 2). Among them, neurological disease consisted of movement disorders (237 genes, 1.65E-13), disorder of basal ganglia (178 genes, 7.88E-13), neuromuscular disease (190 genes, 5.69E-11), neurological signs (151 genes, 6.19E-10), dyskinesia (143 genes, 1.34E-09), Huntington's disease (135 genes, 1,44E-09), progressive motor neuropathy ( 97 genes, 7.78E-06), damage of brain (31 genes, 1.25E-04), seizures (77 genes, 2.22E-04), hypomyelination (11 genes, 4.76E-04), damage of cerebral cortex (11 genes, 8.94E-04) , dysmyelination of sciatic nerve (4 genes, 9.96E-04), and seizure disorder (84 genes, 1.09E-03) (Fig. 3A). Furthermore, we determined the canonical pathways by IPA tool using genes differentially expressed in cochlear tissues of *Cmah*-null mice. Of them, the pathway classified aging-related functional groups as follow: glutathion-mediated detoxification, protein ubiquitination pathway, phagosome maturation, mitochondrial dysfunction, glutathione redox reaction I, acetyl-coA biosynthesis, oxidative phosphorylation, and TCA cycle for oxidative stress and mitochondrial dysfunction (Group I) and Wnt/β-catenin signaling (Group II) (Fig. 3B). Taken together, these results provide further information for evaluation of the potential effects and altered functions caused by *Cmah* gene disruption.

**Table 1:** KEGG pathway analysis in the cochlear tissues of *Cmah*-null mice

Term	Count	P value	Genes
Oxidative phosphorylation	47	3.47E-19	*Uqcrc2, Ndufb3, Atp5e, Ndufb4, Ndufb5, Ndufb8, Ndufb9, Cyc1, Cox7b, LOC100048613, Atp6v1g2, Atp12a, Ndufb2, Ndufs6, Ndufs4, Ndufs8, Cox6b1, Cox6b2, Ndufs3, Cox17, Ndufs2, Atp5h, Ndufa4, Ndufa5, Ndufa2, Atp5j2, Ndufa3, Cox7a2, Ndufa8, Cox8b, Cox7a1, Ndufa9, Ndufa6, Ndufa7, Ndufc2, Atp5f1, Ndufc1, Ndufa1, Cox6c, Sdhb, Atp6v1a, Uqcrh, Ndufv2, Sdhd, Atp5c1, Atp6v0a1, Atp5a1*
Huntington's disease	54	1.54E-17	*Uqcrc2, Atp5e, Pparg, Cyc1, LOC100048613, LOC100046796, Ndufs6, Bdnf, Ndufs4, Ndufs8, Ndufs3, Atp5h, Ndufs2, Cycs, Ndufc2, Ndufc1, Ppargc1a, Dctn1, Cox6c, Uqcrh, Atp5c1, Ndufb3, Ndufb4, Polr2g, Ndufb5, Ndufb8, Ndufb9, Cox7b, Ndufb2, Dnali1, Cox6b1, Cox6b2, Hap1, Ndufa4, Trp53, Ndufa5, Ndufa2, Cox7a2, Ndufa3, Cox7a1, Cox8b, Ndufa8, Ndufa9, Ndufa6, Creb1, Ndufa7, Atp5f1, Ucp1, Ndufa1, Sod2, Sdhb, Ndufv2, Sdhd, Atp5a1*
Parkinson's disease	42	9.64E-15	*Uqcrc2, Ndufb3, Atp5e, Ndufb4, Ndufb5, Ndufb8, Ndufb9, Cyc1, Cox7b, LOC100048613, Ndufb2, Ndufs6, Ndufs4, Ndufs8, Cox6b1, Cox6b2, Ndufs3, Ndufs2, Atp5h, Ndufa4, Ndufa5, Ndufa2, Ndufa3, Cox7a2, Ndufa8, Cox8b, Cox7a1, Ndufa9, Ndufa6, Ndufa7, Cycs, Ndufc2, Atp5f1, Ndufc1, Ndufa1, Cox6c, Sdhb, Uqcrh, Ndufv2, Sshd, Atp5c1, Atp5a1*
Alzheimer's disease	48	1.72E-13	*Ndufb3, Uqcrc2, Atp5e, Ndufb4, Ndufb5, Ndufb8, Ndufb9, Cyc1, Cox7b, LOC100048613, Ndufb2, Ndufs6, Ndufs4, Ndufs8, Cox6b1, Il1b, Cox6b2, Ndufs3, Ndufs2, Atp5h, Ndufa4, Ndufa5, Ndufa2, Ndufa3, Cox7a2, Ndufa8, Cox8b, Cox7a1, Ndufa9, Ndufa6, Ndufa7, LOC100048622, Cycs, Ndufc2, Atp5f1, Ndufc1, Ndufa1, Cox6c, Ncstn, Sdhb, Uqcrh, Atp2a3, Ndufv2, Sdhd, Calm4, Atp5c1, Atp5a1, Calm2*
Fatty acid metabolism	17	1.80E-07	*Cpt1b, Acaa2, Acadsb, Acadm, Cpt2, Acads, Adh5, Echs1, Acat2, Acadl, Peci, Dci, Hadhb, Acadvl, Acsl1, Adh1, Acsl5*
Citrate cycle (TCA cycle)	13	2.25E-06	*Sdhb, Idh3g, Aco2, Suclg1, Dld, Sdhd, Idh2, Idh1, Pdha1, Fh1, Pdhb, Pck1, Mdh1*
Cardiac muscle contraction	15	0.003403691	*Uqcrc2, Cacna2d1, Actc1, Cox7a2, Cox8b, Cox7a1, Atp1b2, LOC100048613, Cox7b, Cyc1, Cacng5, Cox6c, Uqcrh, Cox6b1, Cox6b2*
Glycolysis / Gluconeogenesis	13	0.007452722	*Pfkp, Adh5, Pfkm, Fbp2, Pdhb, Aldh3a1, Pck1, Pgm2, Tpi1, Adh1, Dld, Eno3, Pdha1*
Glutathione metabolism	10	0.021733591	*Mgst3, Gstm1, Gpx4, Gpx3, Idh2, Gstz1, Idh1, Anpep, Gclm, Mgst1*
Proteasome	9	0.032475591	*Psmb5, Psmb7, Psmd14, Psmb6, Psma4, Psmb3, Shfm1, Psme4, Psmd6*
Wnt signaling pathway	12	0.046687622	*Trp53, Wnt10a, Fzd10, Ccnd1, Wnt7b, Wnt4, Btrc, LOC100048622, Wnt6, Axin2, Prkx, Nfatc1*

Number of the observed differentially expressed genes in the category. P values is less than 0.05 (P<0.05).

**Table 2:** Disease and disorders identified by Ingenuity Pathway Analysis in the cochlear tissues of *Cmah*-null mice

Diseases and Disorders		
Name	P value	#Molecules
Neurological Disease	9.49E-14~1.75E-03	385
Psychological Disorders	5.14E-13~1.30E-09	178
Skeletal and Muscular Disorders	2.99E-10~1.60E-03	472
Infectious Disease	4.20E-10~1.58E-03	298
Hereditary Disorder	1.30E-09~1.60E-03	163

P values represent significance of overrepresentation of candidate genes within respective gene groups. P value ranges indicate values for various disease subclassifications (not shown).

### Alteration of genes involved in oxidative damage and ROS metabolism

To clarify differential mRNAs expression of oxidative stress and antioxidant defense-related genes, we examined the expression of genes related to oxidative damage and ROS metabolism pathway in the cochlear tissues from WT- and *Cmah*-null mice, using a pathway-focused PCR array. RT-qPCR analysis revealed a significant down-regulation in most of gene expressions in *Cmah*-null mice with AHL, except one gene, *Apoe* (Table 3). Down-regulated genes consisted of 3 specific subsets for oxidative damage and dysregulated ROS metabolism as follows: 1) glutathion peroxidases (*Gpxs*), peroxiredoxins (*Tpxs*), peroxidases, and antioxidant-related genes, 2) superoxide dismutases (*Sod*), genes involved in superoxide metabolism, genes involved in ROS metabolism, and oxidative damage responsive genes for ROS metabolism, and 3) oxygen transporters. Therefore, these results suggested specific subsets of oxidative stress genes that appear to play an important role in AHL in the *Cmah*-null mice.

**Table 3:** Alteration of genes involved in oxidative damage and ROS metabolism in the cochlea of *Cmah*-null mice

Subsets	Groups	Genes
Antioxidants	Glutathion Peroxidases (*Gpxs*)	*Gpx1* (−45.63), *Gpx2* (−4.04), *Gpx7* (−5.55)
	Peroxiredoxins(*Tpxs*)	*Prdx1* (−24.50), *Prdx4* (−107.49)
	Peroxidases	*Cat* (−5.59), *Duox1* (−22.85), *Epx* (−43.83), *Lpo* (−158.02), *Mpo* (−5.61), Ptgs2 (−68.74), *Rag2* (−101.27), *Serpinb1b* (−88.65), Tpo (−1114.36)
	Antioxidants	*Alb*(−3650.81), Sod1(−31.69), *Srxn1*(−4.20), *Txnrd2*(−37.61), *Txnrd3*(−37.40)
ROS metabolism	Superoxide Dismutases (*Sod*)	Sod1(−31.69), Sod2(−53.56)
	Genes involved in superoxide metabolism	*Cygb* (−4.12), *Ncf1* (−15.17), Ncf2 (−26.17), *Nos2* (−216.32), *Nox1* (−685.97), *Nox4* (−59.26), *Noxa1* (−3891.20), *Recql4* (−42.05), *Scd1* (−8.50)
	Genes involved in ROS metabolism	*Fmo2* (−5.95)
	Oxidative Damage Responsive Genes	Als2 (−10.64), *Apoe* (12.54), *Cat* (−5.59), *Ccl5* (−51.16), *Duox1* (−22.85), *Epx* (−43.83), *Ercc2* (−9.90), *Ercc6* (−9.77), *Duox1* (−55.87), *Gclc* (−18.80), *Gpx1* (−45.63), *Gpx2* (−4.04), *Gpx7* (−5.55), *Hmox1* (−19.82), *Hspa1a* (−22.78), *Krt1* (−5.23), *Mpo* (−5.61), *Nqo1* (−7.81), *Prdx1* (−24.50), *Psmb5* (−7.16), *Sod1* (−31.69), *Tpo* (−1114.36), *Txn1* (−99.46), Txnrd2 (−37.61), *Ucp3* (−41.41), *Xpa* (−19.89)
Oxygen Transporters		*Atr* (−9.36), Cygb (−4.12), *Dnm2* (−6.47), *Fancc* (−10.66), *Ift172* (−4.69), *Mb* (−14.77), *Ngb* (−257.96)

### Molecular mechanisms of sirtuins and oxidative stress regulation, and Wnt signaling that involved in *Cmah*-null mice with hearing loss

**Figure 4:**
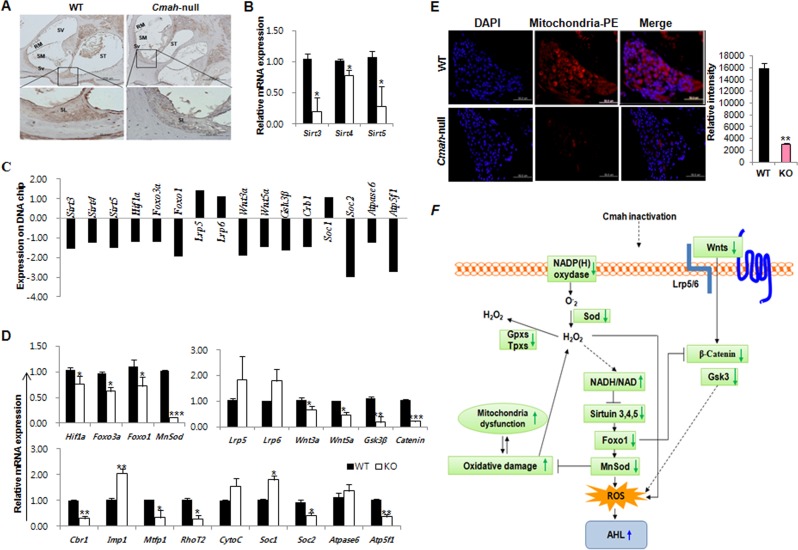
Molecular mechanisms of sirtuins, oxidative stress regulation, and Wnt signaling involved in *Cmah*-null mice (**A**) IHC in cochlea of WT- and *Cmah*-null mouse for detection of Sirt3. Bar: 200 um. SV: scala vestibule, RM: reissner's membrane, SM: scala media, ST: scala tympani, Sv: stria vascularis, SL: spiral lamina. (**B**) Expression levels of sirtuins were determined by RT-qPCR of RNA samples from cochlear tissues of WT- and *Cmah*-null mice. (**C**) Expression of AHL related genes on the microarray data from cochlear tissues of *Cmah*-null mice. (**D**) Expression levels of genes involved in the regulatory factor of sirtuin, Wnt signaling, and mitochondrial functional regulation were determined by RT-qPCR of RNA samples from cochlear tissues of WT- and *Cmah*-null mice. (**E**) Measurement of mitochondrial activity in the cochlea of WT- and *Cmah*-null mice. Immunofluorescence was performed using a mitochondrial marker antibody. The intensity of the fluorescent signal indicates mitochondria numbers or the level of integrity. (**F**) Summary model describing the molecular mechanisms for sirtuins and oxidative stress regulation, Wnt signaling, and mitochondrial dysfunction by identified RT-qPCR results and array date set. The down-regulated Sirt3 lead to oxidative damage and mitochondrial dysfunction and could be alter various signaling pathways in *Cmah*-null mice with AHL. *Sirt3-5*: NAD-dependent protein deacetylase sirtuin3-5, *Hif1α*: Hypoxia-inducible factor 1-alpha, *Foxo3a*: Forkhead box O3, *Foxo1*: Forkhead box protein O1, *MnSod*: superoxide dismutase 2, mitochondrial, *Lrp5-6*: Low-density lipoprotein receptor-related protein 5-6, *Wnt*: *Wingless*, the *Drosophila melanogaster* segment-polarity gene, and *Integrase-1* the vertebrate homologue, *Gsk3β*: Glycogen synthase kinase 3 beta, *Cbr1*: carbonyl reductase 1, *Imp1*: inner mitochondrial membrane peptidase-like, *Mtfp1*: mitochondrial fission process 1, *RhoT2*: Mitochondrial Rho GTPase 2, *CytoC*: Cytochrome C, *Soc1-2*: soluble oxidase component 1-2.

To address the underlying mechanisms of AHL in *Cmah*-null mouse, we analyzed the expression of their genes that involved sirtuins, oxidative stress regulation, Wnt signaling, mitochondrial dysfunction identified by pathway analysis on microarray and a pathway-focused PCR array. As shown in Fig. 4A, Sirt3 protein expression was significantly reduced in cochlear tissues of *Cmah*-null mouse, compared to their control. Also, RT-qPCR analysis showed that *Sirt3, 4*, and *5* gene expressions is significantly reduced in *Cmah*-null mice than in controls (Fig. 4B). To determine the expression of AHL related genes in the cochlear tissues of *Cmah*-null mouse, we checked the expression profiles of these genes on the microarray analysis (Fig. 4C). The result shows that the data sets obtained from the microarray analysis accurately matched with RT-qPCR analysis in *Cmah*-null mouse (Fig. 4C and D). Like that, expressions of *Hif1α* and *Foxo3a* mRNAs as transcription factor regulated by *Sirt3* as well as *Foxo1* and *MnSod* mRNAs expressions as downstream regulator of *Sirt3* was decreased in *Cmah*-null mice (Fig. 4D).

To observe the regulatory factors in Wnt signaling, which might control by ROS in *Cmah*-null mouse, we analyzed the expression of Wnt signaling-related genes (*Lrp5, Lrp6, Wnt3a, Wnt5a, Gsk3β*, and *Catenin*). The result shows that Wnt signaling-related gene expressions in *Cmah*-null mouse were significantly decreased, whereas the Wnt receptor gene expression was not altered (Fig. 4D). Next, we examined mitochondrial dysfunction-related gene expression. The expression of these genes (*Crb1, Mtfp1, RhoT2, Soc2*, and *Atp5f1*) with a role as regulators and mediators of mitochondrial molecular transport was significantly down-regulated (Fig. 4D). We therefore investigated whether altered genes affect the mitochondria expression in sensory neurons of *Cmah*-null mouse by immunofluorescence. As shown in Fig. 4E, mitochondrial positive signals were significantly decreased in *Cmah*-null mice, compared to control mice. Taken together, we proposed a key underlying mechanism for explaining AHL in *Cmah-*null mice (Fig. 4F), suggested that AHL in *Cmah*-null mice was caused by down-regulation of *Sirt3* mRNA expression, which resulted in histological abnormalities in vestibular otoconial epithelia and the cochlear sensory epithelium, and neuronal cell loss in inner ears due to mitochondrial dysfunction by excessive oxidative stress.

## DISCUSSION

In this study, *Cmah*-null mice showed AHL. To identify a change by hearing loss with aging in *Cmah*-null mice, we have analyzed global gene expression profiling, gene interaction network, and signaling pathway using Illumina Beadchip microarray in inner ear of WT- and *Cmah*-null mice. *Cmah*-null mice with hearing loss are closely associated with neurological disease (Alzheimer's disease, Parkinson's disease and Huntington's disease), psychological disorders, skeletal and muscular disorders, infectious disease, and hereditary disorder. These observations are consistent with previous knowledge of AHL, which shows Alzheimer's disease, Parkinson's disease, and Huntington's disease [31]. Also, we have identified stress response and Wnt signaling abnormality, and mitochondrial dysfunction, which are up- and down-regulated by the loss of Cmah activity. Especially, we identified a molecular mechanism of oxidative stress regulated by Sirt3 expression, which lead to mitochondrial dysfunction by ROS, in *Cmah*-null mice. Therefore, we concluded that Sir3 dysfunction in *Cmah*-null mice altered various signaling pathways, which are responsible for the most common sensory disorder in the elderly.

Generally, AHL is closely associated with an impaired inner ear, which generally shows loss of sensory hair cells and/or stria vascularis degeneration in the cochlea and neuronal loss (spiral ganglion neurons) in brain [32]. It is generally well-known that GFAP is a neural injury marker or astrocyte marker; NF for an axonal injury marker, VIM for a neural precursor cells marker in CNS, and NSE for neuronal marker. To this end, we studied the alteration of GFAP, NSE, NF, and VIM in cochlear tissues during different stages of AHL used *Cmah*-null mice mimicking AHL. Immunohistochemically there is combined loss of staining for GFAP, NF, and VIM in cochlear tissues of *Cmah*-null mice, whereas NSE staining patterns was optically not changed (Fig. 1). However, all of *Gfap, Nse, Nf*, and *Vim* mRNA expressions in *Cmah*-null mice are significantly down-regulated, compared to control. Of note, cochlear tissues of *Cmah*-null mice showed extensive apoptosis, whereas control mouse showed a moderate apoptosis. Our results suggested that there was a potential astrocyte regenerative response to neuron degeneration in the adult cochlear tissues during the onset and progression stages of AHL-like disease. Therefore, the regenerative responses in the adult cochlear tissues of AHL-like mice may be a potential pathway in attempting to repair the degenerated neurons and restore the dysfunctional neural circuitry.

Having identified differentially expressed genes between *Cmah*-null and control mice, we searched for differential gene expression profiles contributing to hearing loss with aging. In this study, we determined the 11 most significantly enriched pathways with differentially expressed genes identified on microarray analysis by KEGG pathway analysis to explore their functional role. Remarkably, most of them are associated with the oxidative stress, metabolism and degenerative nerve diseases, supporting the pathogenesis hypothesis of age-related dysfunction (Table 1). Previous studies suggest that in case of AHL, oxidative stress is playing significant role. ROS generated in cochleae increase high-intensity noise [33]. Mice lacking antioxidant enzymes demonstrate enhanced age-related cochlear hair cell loss and susceptibility to noise-induced hearing loss [34, 35]. Moreover, oxidative stress increases protein damage, which directly related to age in the cochleae of CBA mice [36]. To address the possibility that in *Cmah*-null mice the cochlear tissues generates excessive ROS, we examined gene expressions that involved in oxidative stress and antioxidant defense using a pathway-focused PCR array. We found that expression of antioxidant and ROS metabolism related genes was significantly down-regulated and/or dysregulated in the cochlear tissues of *Cmah*-null mouse. These observations suggested that oxidative stress in the cochlear tissues of *Cmah*-null mouse played an important role in the pathogenesis of AHL.

In this study, we found that Wnt signaling (*Lrp5, Lrp6, Wnt3a, Wnt5a, Gsk3β*, and *Catenin*)- and mitochondrial molecular transport (*Crb1, Mtfp1, RhoT2, Soc2*, and *Atp5f1*)-regulator gene expressions in the cochlear tissues of *Cmah-*null mice with AHL were significantly down-regulated. Previous studies suggest that Wnt/β-catenin signaling play an important role in critical hepatic development, regeneration, and carcinogenesis [37-39]. The data from these studies suggested that β-catenin regulate hepatic energy metabolism and mitochondrial function [40]. Further, studies from mammalian cells and *Caenorhabditis elegans* showed that β-catenin and Foxos expression in response to oxidative stress have a cross-talk mechanism [41, 42]. Taken our data with previous observations, ROS accumulations in the cochlear tissues of *Cmah*-null mouse might be caused by reduction of both antioxidant- and Wnt/β-catenin signaling-related gene expressions, which ultimately induced mitochondrial dysfunction (Table 3 and Fig. 4). In conclusion, these findings suggest that Cmah disruption could affect a variety of signaling pathways accompanying orchestrated gene expression changes. To the best of our knowledge, our results for the first time showed that down-regulated β-catenin expression in Wnt signaling give rise to ROS in the cochlear tissues of *Cmah-*null mice with AHL (Table 3).

Finally, we found that *Hif1α* and *Foxo3a* mRNAs as well as *Foxo1* and *MnSod* mRNAs expressions, which located in downstream of *Sirt3* gene expression, is significantly decreased. Also, we found that *Sirt3* mRNA expression in the cochlear tissues of *Cmah*-null mouse is significantly down-regulated (Fig. 4). It is well known that Sirt3 are localized in mitochondria and that Sirt3 is the primary deacetylase, which plays an important role in energy production, metabolism, apoptosis, and cell signaling during aging [43-45]. Further, recent several studies also showed that Sirt3 is as an essential player to enhance glutathione antioxidant defense system in mitochondria under CR conditions and demonstrated that Sirt3-dependent mitochondrial adaptations may be a central mechanism of aging retardation in mammals [29, 46, 47]. These observation suggested that ROS accumulations in the cochlear tissues of *Cmah-*null mice with AHL down-regulated Sirt3 expression. As a result, Sirt3 dysregulation leads to oxidative damage and mitochondrial dysfunction (Fig. 4). Therefore, this study could provide a possible mechanism of Sirt3 in ROS and oxidative stress induced metabolism in *Cmah*-null mouse. Based on our results and previous reports, we suggested a hypothetical model that down-regulated Sirt3 lead to oxidative damage and mitochondrial dysfunction and could be alter various signaling pathways in *Cmah*-null mice with AHL. In conclusion, the present study suggests that mice with Cmah deficiency serve an important model for studying hearing loss with aging caused by evolutionary loss of Neu5Gc synthesis in human.

Of course, mice studies are not complementary to human. Furthermore, we cannot expect that mice with a human-like deficiency of Neu5Gc will mimic all consequences of Neu5Gc loss in a hominid ancestor. In this study, we selected *Cmah*-null mice as a model system to represents a state of at least partial characterization of biochemistry, physiology and metabolism and it could serve as a model system for studying hearing loss with aging caused by evolutionary loss of Neu5Gc synthesis in human. However many additional studies are required to prove hearing loss with aging caused by evolutionary loss of Neu5Gc synthesis in human.

## METHODS

### Animal ethics

All animal experiments were approved and performed under the guidelines of the Konkuk University Animal Care and Experimentation Community [IACUC approval number: KU12045]. *Cmah* <tm1Ykoz> knockout mice were kindly provided by RIKEN Center for Developmental Biology (CDB, Japan).

### Immunohistochemistry (IHC)/ immunofluorescence

For histology, inner ears were harvested after intracardiac perfusion with 4% paraformaldehyde, postfixed overnight, decalcified in 8% EDTA, and embedded in paraffin. Sections were stained with Hematoxylin QS for light microscopy. For IHC, the cochlear tissues were fixed with 4% paraformaldehyde and then embedded on slides. After deparaffination and rehydration in PBS, sections were blocked in background Sniper solution. After washing, the samples were incubated with specific primary antibodies for Neu5Gc (1:200 dilution; Sialix, San Diego, CA, USA), GFAP, NSE, NF, VIM (prediluted: Leica Biosystems Inc. Buffalo Grove, IL, USA), and Sirt3 (1:200 dilution; Santa Cruz Biotehnology, Inc. Dallas, TX, USA) at 4°C overnight. After the incubation, the samples were washed and incubated with horseradish peroxidase-conjugated secondary antibody. Samples were then stained with ImmPACT^TM^ DAB peroxidase substrate (Vector Laboratories; CA, USA) to visualize the signal. Samples were also stained with Hematoxylin QS to provide background information for reference. The samples were mounted using VECTORSHIELD HardSet mounting medium (Vector Laboratories; CA, USA) and observed using fluorescence microscopy (Olympus; Japan). For immunofluorescence, the samples were incubated with primary antibodies for mitochondrial marker (1:1000 dilution, Abcam, Cambridge, UK) at 4°C overnight. After incubate with Alexa Fluor 488-labeled goat anti-mouse (1:500), the samples were mounted using VECTORSHIELD HardSet mounting medium with DAPI (Vector Laboratories) and observed using fluorescence microscopy (Olympus; Japan).

### Apoptotic cell staining

The terminal deoxynucleotidyl transferase–mediated nick end labeling (TUNEL) method was employed for detection of apoptosis using the in situ cell detection kit, tetramethylrhodamine red (TMR, red fluorescence) from Roche Applied Science (Lewes, East Sussex, UK) according to the manufacturer's instructions. For detection of the characteristic chromatin condensation and nuclear fragmentation associated with apoptosis, the samples were mounted using VECTORSHIELD HardSet mounting medium with DAPI (Vector Laboratories) and observed using fluorescence microscopy (Olympus; Japan).

### Microarray analysis of gene expression

WT- and *Cmah*-null mice with same age (12 months) and genetic background (C57BL/6J) were used (n=3 per each group) for microarray analysis. Total RNA was extracted and purified from the cochlear tissues of WT- and *Cmah*-null mice using Trizol and RNeasy columns (Qiagen; Valencia, CA, USA) according to the manufacturer's protocol. The RNA quality was verified using an Agilent Bioanalyzer 2100 (Agilent Technologies; Palo Alto, CA, USA) using the RNA 6000 Pico Assay. Generation of double-stranded cDNA, preparation and labeling of cRNA, hybridization to Mouse Ref-8 v2.0 Expression BeadChip (Illumina, Inc.; San Diego, CA, USA), washing, and scanning were all performed according to the standard Illumina protocol. Arrays were scanned using the Illumina Bead Array Reader Confocal Scanner. Microarray data have been deposited in NCBI's Gene Expression Omnibus and are accessible through GEO Series accession number GSE70659 (Gene expression profile in the cochlear tissue of *Cmah*-null mouse).

### *In silico* data analysis

Differentially expressed genes (DEG) were subjected to intensive investigation to identify their biological functions. GO analysis was performed as described using DAVID (http://david.abcc.ncifcrf.gov) to study the biological function of the regulated genes [48]. The differentially expressed genes were compared to all of the genes on the microarrays and a P value computed using a Fisher's exact test. KEGG is a collection of online databases dealing with genomes, enzymatic pathways, and biological chemicals [49]. The KEGG PATHWAY Database records networks of molecular interactions in the cell that includes organism-specific network maps (http://www.genome.jp/kegg/). In addition, canonical pathways and functions analyses were created by IPA (Ingenuity Systems, Mountain View, CA, USA), which assists with microarray data interpretation via grouping DEG into known functions, pathways and gene interaction networks, based primarily on human and rodent studies. Significance was set at a P value of 0.05.

### Pathway-focused PCR array

The Mouse Oxidative Stress and Antioxidant Defense RT^2^ Profiler™ PCR array (SABiosciences Corp., Valencia, CA) was used to measure the alteration of gene expression in the cochlear tissues from WT- and *Cmah*-null mice. The RT^2^ Profiler™ PCR Array (SABiosciences Corp.) is a commercially available set of optimized RT-qPCR primer assays on a 96-well plate. Total RNA was then treated with DNase I, reverse-transcribed using an RT^2^ First Strand Kit (Qiagen), and brought to a final volume of 120 μL. cDNA from individual samples was used as a template for the PCR array, according to the array instructions, using SYBR green on an ABI ViiA^TM^ 7 system (Applied Biosystems). Data were analyzed using SABiosciences RT2 Profiler PCR Data Analysis software, available at http://pcrdataanalysis.sabiosciences.com/pcr/arrayanalysis.php, and were considered significant at >4-fold change. Relative quantitation for each gene was determined by normalization to 5 housekeeping genes (*Actb, B2m, Gapdh, Gusb*, and *Hsp90ab1*), comparing the WT and *Cmah*-null groups by using the 2-ΔΔCt method.

### Quantitative real-time polymerase chain reaction (RT-qPCR)

The total RNA obtained from the cochlea of WT- and *Cmah*-null mice was reverse-transcribed with the QuantiTect Reverse Transcription Kit (Qiagen; Valencia, CA, USA) as recommended by the manufacturer. To evaluate gene expression, RT-qPCR was performed on an ABI ViiA^TM^ 7 system (Applied Biosystems; Foster City, CA, USA) with SYBR Green as the fluorescence detection method (Bio-Rad; Hercules, CA, USA). The mouse *Gadph* gene was used as an internal control to normalize the RT-qPCR efficiency and for quantification of gene expression (Supplementary Table 1 and 2). The RT-qPCR was performed in triplicate for each sample.

### Statistical analysis

All experimental data was presented as means ± standard deviation (SD). Each experiment was performed at least three times. For statistical analysis (Fig. 1 and 4), one-way analysis of variance (ANOVA) was performed to determine whether there were differences among the groups, and Fisher's post-test was performed to determine significance between pairs of groups. In all experiments, *P < 0.05, **P < 0.01, and ***P < 0.001 were considered significant.

## SUPPLEMENTARY INFORMATION FIGURE AND TABLES



## Abbreviations

AHL: age-related hearing loss
Sirt3: sirtuin3
Cmah: CMP-N-acetylneuraminic acid hydroxylase
ROS: reactive oxygen species
KEGG: Kyoto Encyclopedia of Genes and Genome
DAVID: Database for Annotation, Visualization and Integrated Discovery
WT: wild-type
Hif1α: hypoxia-inducible factor 1-alpha
Foxo3a: forkhead box O3a
Foxo1: folkhead box protein O
MnSod: mamganese superoxide dismutase 2
RT-qPCR: quantitative real-time polymerase chain reaction
SV: scala vestibule
SM: scala media
ST: scala tympani
SG: spiral ganglion
BM: basilar membrane
RM: reissner's membrane
Sv: stria vascularis
OT: otoconia
OM: otolithic membrane
NHC & SC: neuroepithelial hair cells and supporting cells
HC: hair cells
OHCs: outer hair cells
SL: spiral lamina.
