# Supplementary material for: Oxidative stress and ROS metabolism via down-regulation of sirtuin 3 expression in Cmah-null mice affect hearing loss
Source: Aging (Albany NY). 2015 Aug 29;7(8):579–92. doi: 10.18632/aging.100800 (PMC4586103; doi:10.18632/aging.100800)
Supplement: Supplementary file 1 [file aging-07-579-s001.pdf]

SUPPLEMENTARY INFORMATION

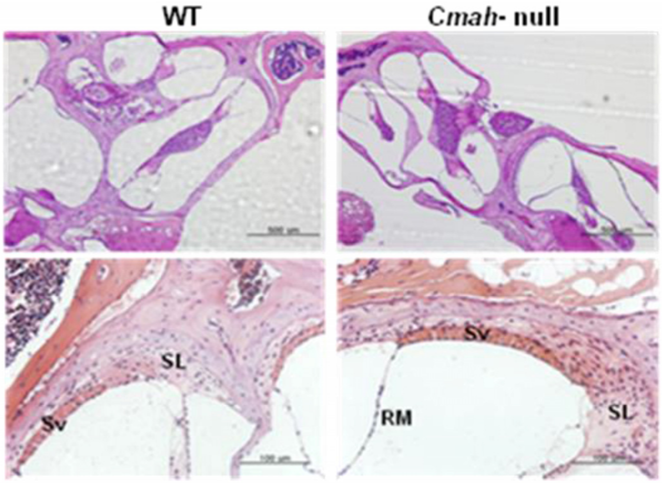

Supplementary Figure 1. Histological analysis in inner ear of WT- and *Cmah*-null mouse of 5 week-old. SL: Spiral lamina, Sv: Stria vascularis, RM: Reissner's membrane.

Supplementary Table 1. Primer sets for neuronal cell marker

Genes	Forward primer	Reverse primer	Product (bp)
Gfap	AGAAAACCGCATCACCATTC	TCACATCACCACGTCCTTGT	184
Nse	AGCCCTCATCAGCTCAGGTA	CTGAGCAATGTGGCGATAGA	184
Nf	GCCGAAGAGTGGTTCAAGAG	TGTCTGCATTCTGCTTGTCC	205
Vim	ATGCTTCTCTGGCACGTCTT	AGCCACGCTTTCATACTGCT	206

Supplementary Table 2. Primer sets used for identify molecular mechanisms of sirtuins, oxidative stress regulation, and Wnt signaling

Genes	Forward primer	Reverse primer	Product (bp)
Sirt3	TACAGGCCCAATGTCACTCA	ACAGACCGTGCATGTAGCTG	167
Sirt4	CGAGCAAAAGCTCCCAATAG	GTTCATTTCCAGCCTTTGGA	151
Sirt5	CCCAGAGCCAGAGACTCAAG	CAGAGGATGTTCCCACCACT	191
Hif1α	GCAGCAGGAATTGGAACATT	GCATGCTAAATCGGAGGGTA	150
Foxo3a	GGGGAGTTTGGTCAATCAGA	GCCTGAGAGAGAGTCCGAGA	193
Foxo1	GTGAACACCATGCCTCACAC	CACAGTCCAAGCGCTCAATA	211
MnSod	GGCCAAGGGAGATGTTACAA	CCTTGGACTCCCACAGACAT	213
Lrp5	GTGTGCAGTTGCAGGACAAT	CTCCAGGGGATCGTAGTCAA	176
Lrp6	GACAGACTGGGGAGAAGTGC	AACGTCAAGGCAAAAGGATG	234
Wnt3a	ATGGCTCCTCTCGGATACCT	GGGCATGATCTCCACGTAGT	192
Wnt5a	CAAATAGGCAGCCGAGAGAC	CTCTAGCGTCCACGAACTCC	217
Gsk3β	CAGTGGTGTGGATCAGTTGG	ATGTGCACAAGCTTCCAGTG	232
Catenine	ACAGGGTGCTATTCCACGAC	CTGCACAAACAATGGAATGG	217
Cbr1	AGTGGTGAATGTGTCCAGCA	CAGGACTGTCACCCCAATCT	211
Imp1	GGCATCCAAAGAGGTGACAT	ACATGACCTGTTGGCACGTA	155
Mtfp1	GCTGTGGTGTGGTTGAGCTA	ACACAGACGGTTGATGGTGA	198
RhoT2	CAGTTACCCGCGAGAAGAAG	GGCTGTCAGCATTCACTTCA	240
CytoC	TTCCACAACCCTCATGTGAA	TAAGGGTCCAAAACCAGTGC	109
Soc1	AGTGGCCTTGAAAGAAAGCA	GTAGCCTGCCCTTGCTATTG	79
Soc2	CCTTCGCTGAACTTGTCCTC	CCCTAGAGCCAGTAGCATCG	100
Atpase6	CCTTCCACAAGGAACTCCAA	CCTTCCACAAGGAACTCCAA	187
Atp5f1	TCAGAAGCGCCATTACCTCT	TTGGCAATGGTCTCCTTTTC	234
